# Two ways to fold the genome during the cell cycle: insights obtained with chromosome conformation capture

**DOI:** 10.1186/1756-8935-7-25

**Published:** 2014-11-25

**Authors:** Job Dekker

**Affiliations:** Program in Systems Biology, Department of Biochemistry and Molecular Pharmacology, University of Massachusetts Medical School, 368 Plantation Street, Worcester, MA 01605-0103 USA

**Keywords:** Chromatin looping, Chromosome conformation capture, Chromosome folding, Epigenetic inheritance, Mitotic chromosome, Nucleus

## Abstract

Genetic and epigenetic inheritance through mitosis is critical for dividing cells to maintain their state. This process occurs in the context of large-scale re-organization of chromosome conformation during prophase leading to the formation of mitotic chromosomes, and during the reformation of the interphase nucleus during telophase and early G1. This review highlights how recent studies over the last 5 years employing chromosome conformation capture combined with classical models of chromosome organization based on decades of microscopic observations, are providing new insights into the three-dimensional organization of chromatin inside the interphase nucleus and within mitotic chromosomes. One striking observation is that interphase genome organization displays cell type-specific features that are related to cell type-specific gene expression, whereas mitotic chromosome folding appears universal and tissue invariant. This raises the question of whether or not there is a need for an epigenetic memory for genome folding. Herein, the two different folding states of mammalian genomes are reviewed and then models are discussed wherein instructions for cell type-specific genome folding are locally encoded in the linear genome and transmitted through mitosis, e.g., as open chromatin sites with or without continuous binding of transcription factors. In the next cell cycle these instructions are used to re-assemble protein complexes on regulatory elements which then drive three-dimensional folding of the genome from the bottom up through local action and self-assembly into higher order levels of cell type-specific organization. In this model, no explicit epigenetic memory for cell type-specific chromosome folding is required.

## Review

Chromosome organization and nuclear organization have been studied for many years using microscopic and, more recently, molecular approaches [1–8]. Understanding how cells organize their genome inside the cell nucleus is important given its relation to genomic activities including gene regulation [5, 9–11], DNA repair [12–14], and transmission of chromosomes to daughter cells [15–18]. It has long been recognized that nuclear and chromosome organization, i.e., where genes are spatially located with respect to each other and with respect to nuclear landmarks such as the nuclear envelope, is related to gene activity and chromatin status and is often cell type-specific (e.g., [2]). Further, ever since chromosomes were first observed in the late 19th century, it is known that they change their appearance during the cell cycle, from a decondensed state in interphase to a highly condensed and reproducible structure during mitosis [19]. Major questions in the field are what structural principles underlie these different chromosome organizations, which features are cell type-specific, and how these structures are contributing to cell type-specific gene regulation. Understanding how chromosomes are organized in different cells and across the cell cycle is interesting by itself, but it may also shed light on a basic question at the heart of epigenetics, which is whether and how information regarding cell types and gene expression patterns can be stably transmitted through mitosis and whether any cell type-specific chromosome organizational features are, or need to be, inherited.

Cell type is to a large extent reflected in, and driven by, the set of genes that a cell expresses. Gene expression patterns are determined by the activity of proximal gene promoters and distal elements such as enhancers. The activity of promoters and enhancers is in turn reflected in the cell type-specific locations of open chromatin regions where transcription factors bind and that are further marked by local and regional patterns of a wide array of histone modifications [20, 21]. As a result, cell type is closely correlated to chromatin state throughout the genome [22–25].

**Figure 1:**
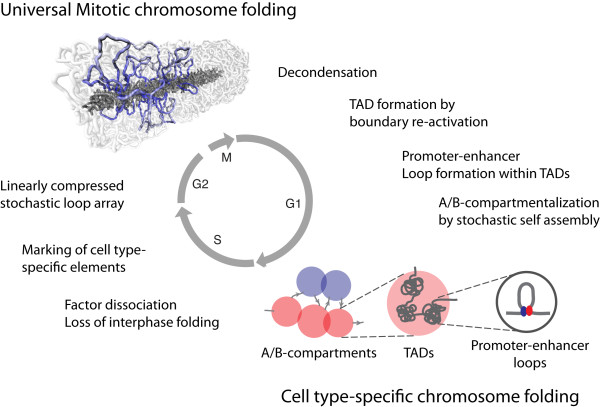
**Proposed model for genome folding dynamics during the cell cycle.** In interphase genome folding is defined by locus-specific compartments and chromatin loops. A/B-compartments and promoter-enhancer loops are cell type-specific, whereas topologically associating domains (TADs) are more tissue-invariant. In prophase many chromatin complexes dissociate from the chromosome, the interphase chromosome organization is lost and replaced by a locus-independent, universal, and cell type-invariant mitotic structure. Mitotic chromosomes form longitudinally compressed stochastically positioned loop arrays. Although mitotic chromosome folding is locus-independent and universal, specific loci, such as TAD boundaries, and cell type-specific elements, such as enhancers, remain marked. In early G1 the mitotic chromosome decondenses again. Next, TAD boundaries are re-activated and TADs are re-established. Subsequently, promoter and enhancer re-associate with transcription factors and other complexes and promoter-enhancer interactions are re-established. At the same time, groups of active and inactive TADs self-assemble into higher order structures corresponding to A- and B-compartments, respectively. This model of the order of events is currently hypothetical and based on theoretical considerations (see text). The figure of the mitotic chromosome was made by Maxim Imakaev, Geoff Fudenberg, Natalia Naumova, and Leonid Mirny.

Enhancers influence expression of genes over large genomic distances (up to hundreds of kb). One mechanism for such long-range gene regulation involves direct physical interactions between promoters and distal regulatory elements [26–30]. Thus, chromatin folds in three-dimensional (3D) structures to enable and control enhancer-promoter communication. Given that promoter and enhancer activity is cell type-specific, many (but not all) aspects of how chromosomes fold are also likely to be highly cell type-specific. For cycling cells to maintain their pattern of gene expression they need to ensure that their daughter cells will continue to express the same set of genes. This requires cells to somehow “remember” which genes and regulatory elements were active and spatially interacting in the previous cell cycle [31]. This memory process must be able to withstand dissociation of transcription factors and RNA polymerase II from many (but not all) sites throughout the genome [32–35] and a dramatic spatial re-organization and condensation of chromosomes during mitosis (Figure 1).

This review will focus on mammalian chromosomes and their spatial organization during the cell cycle. The organization of chromosomes, both in the interphase nucleus and during mitosis, has been studied for many years, and many of the seminal findings in this area have been obtained by microscopic studies during the last several decades. A comprehensive overview of all the important work performed in this area is beyond the scope of this article. Many excellent review articles have been written that cover these studies and the insights and models they provided (e.g., [1–5, 7, 8, 36–38]). Here, the focus will be specifically on recent findings over the last 5 years, obtained by chromosome conformation capture (3C), and these findings will be discussed in the context of earlier studies. The 3C-based methods themselves are not described in detail as they have been covered by several recent reviews [39–41]. After outlining current ideas of how interphase and mitotic chromosomes are organized, it is argued that an understanding of what epigenetic information is stored, and how, inside mitotic chromosomes will not only provide insights into how cells maintain their differentiated state and gene expression profile but will also reveal the set of instructions cells require and the mechanisms they employ to fold their chromosomes in three-dimensions during the subsequent interphase.

### Interphase organization of the 3D genome

From extensive studies using microscopic approaches, and more recently molecular and genomic methods, a detailed view of the 3D arrangement of chromosomes inside interphase nuclei is emerging (Figure 1) [1–8, 38, 41]. Imaging approaches have been instrumental in uncovering many of the critical features of the organization of the interphase nucleus. One characteristic feature is compartmentalization: mammalian chromosomes form a hierarchical organization of nested domains of various types [5]. A second, but related, feature is the colocalization of loci with each other and with nuclear structures such as the nuclear envelope and nucleoli. Another feature that is more readily detectable using 3C-based approaches is the widespread long-range interaction between defined functional elements including looping between gene promoters and distal enhancers.

#### Nuclear and chromosomal compartmentalization

The first level of compartmentalization occurs at the level of the nucleus where individual chromosomes occupy separate territories [1, 8, 42]. Employing Fluorescence *In Situ* Hybridization (FISH) with whole chromosome probes it was observed that chromosomes do not readily mix with other chromosomes. Instead, each chromosome occupies a distinct volume, or territory, in the nucleus [1, 8, 43, 44]. Interestingly, although the positions of specific chromosome territories are stochastic in the population (i.e., are not the same in each cell), they are not random: large chromosomes and gene-poor chromosomes tend to be located near the nuclear periphery, whereas small and gene-rich chromosomes are located more internally (e.g., [42, 43, 45–47]). Locally extensive intermingling of chromosomes can occur where neighboring chromosome territories touch [48]. Employing probes that cover all genes of a chromosome has also shown extensive intermingling of adjacent territories and indicated that these interactions often involve genes [44].

3C-based studies have confirmed the presence of chromosome territories and the preferred association between certain sets of chromosomes. Circularized chromosome conformation capture, or 3C-on-Chip, (4C) and Hi-C experiments have shown that intra-chromosomal interactions are significantly more frequent than inter-chromosomal interactions, even for pairs of loci located tens of Mb apart [49–52]. This observation is consistent with chromosome territory formation. Furthermore, genome-wide Hi-C data has shown that certain sets of chromosomes interact more frequently with each other than with others. For instance, in lymphoblasts, larger chromosomes tend to interact with other larger chromosomes, whereas the smaller and gene-dense chromosomes also interact preferentially [50–52]. These observations are fully consistent with the earlier results obtained by imaging that showed that larger chromosomes are more peripheral and smaller gene-dense chromosomes tend to be more internally positioned in the nucleus [43, 46, 47, 53].

Another well-established level of compartmentalization is the spatial segregation of active and open chromatin (euchromatin) from inactive, closed chromatin (facultative and constitutive heterochromatin). Initially, such separation was observed by imaging, e.g., by electron microscopy (e.g., [54]). Densely staining chromatin, such as centromeric and telomeric heterochromatin, is found near the nuclear envelope and around nucleoli [55–58], and in some cell types and under certain conditions as foci in the nuclear interior (e.g. [59]). Further experiments using immunofluorescence to localize the positions of histone modifications typically associated with either active or inactive chromatin confirmed that these two types of chromatin tend to occupy distinct parts of the nucleus, with inactive chromatin mostly near the periphery and around nucleoli, and active chromatin located more internally (e.g., [60, 61]).

Compartmentalization of active and inactive chromatin domains has also been observed using 3C-based methods. For instance, 4C analyses showed that active genes interact with other active genes throughout the genome [49], whereas inactive genes associate with inactive genes. Genome-wide Hi-C data has shown that chromosomes are composed of large chromatin domains referred to as compartments: active and open chromatin domains preferentially interact with each other to form A-compartments, while inactive and closed chromatin domains form B-compartments [50–52]; these compartments are typically several Mb in size. Where adjacent chromosome territories mingle, a similar preferential homotypic chromatin interaction is observed: interchromosomal interactions are often between gene-dense A-compartments or, less frequently, between B-compartments, but rarely between A- and B-compartments [44, 50, 62].

Compartmentalization of active and inactive chromatin domains is likely driven at least in part by the fact that active and inactive loci interact with specific sub-nuclear structures. For instance, active genes tend to be found co-localized at sub-nuclear sites, sometimes referred to as transcription factories, that are enriched in RNA polymerase II and other transcription- and splicing-related machineries [10, 63–65]. Similarly, inactive chromatin domains are often found associated with the nuclear lamina [57, 58, 66]. Consistent with this, the generally inactive B-compartments identified by Hi-C analysis often overlap with lamin-associated domains identified by DamID, and are frequently found near the nuclear envelope by FISH studies [66].

Some microscopic observations have suggested the existence of another type of chromosomal domain that is smaller than A- and B-compartments. Direct staining of chromatin has led to the identification of chromosomal domains (CDs). These CDs are observed as small bodies of chromatin, probably several hundred kb in size, that move as a unit and correspond to replication domains [1, 67]. 3C-based studies have recently also uncovered the presence of smaller chromosomal domains genome wide. Because 3C-based methods have the potential to detect chromatin structures in the range of 1 to hundreds of kb, a size range that is typically more difficult to analyze by light microscopy, these methods have been instrumental in probing the structure of chromosomes at such a finer scale. Both chromosome conformation capture carbon copy (5C) and Hi-C studies revealed that A- and B-compartments are themselves composed of smaller domains, referred to as topologically associating domains (TADs) or topological domains [68, 69]. TADs are defined as contiguous chromosomal regions that contain loci which interact frequently with each other, but much less frequently with loci outside the domain. In mouse and human cells, TADs are several hundreds of kb up to 1 to 2 Mb in size, much smaller than A/B-compartments. Analysis of multiple cell lines has revealed that TADs are to a large extent tissue invariant [68, 69], although more detailed and higher-resolution studies are needed. This has led to the proposal that they are the fundamental structural building blocks of chromosomes [6, 41, 70]. It is tempting to propose that CDs and TADs are the same entities, although direct proof for this is still lacking.

Two lines of evidence indicate that TADs also represent functional domains. First, genes located within a TAD can be correlated in their expression pattern across differentiation [68]. Second, using a completely independent method based on a functional enhancer trap approach, Symmons et al. found that chromosomal domains influenced by enhancers correspond closely to TADs, indicating that TADs are the target structure of regulatory elements [71].

The mechanisms by which TADs are formed, and the DNA elements that define them, remain largely unknown. TAD boundaries are enriched in a number of genomic features including promoters, CTCF sites, and SINE repetitive elements [69, 72, 73]. Knock down of CTCF results in some loss of TAD boundary activity, albeit modestly [74]. A large fraction of CTCF sites are not located at TAD boundaries, providing further indications that CTCF sites are not sufficient for boundary formation and that additional factors must play roles in defining TADs and their boundaries; one such factor could be the cohesin complex. Removal of this complex leads to relaxation of TADs, including reduction in interactions between loci located within TADs, but again the effect is small [74–76]. Clearly, other complexes play roles.

The physical mechanisms by which large adjacent chromatin masses can remain spatially separated are not understood. One possibility is that domain boundaries correspond to sites attached to some sub-nuclear structure or scaffold. Such associations have been observed before (e.g., [77, 78]), but their relevance remains a topic of discussion in the field. Another, partially related potential mechanism is the formation of large supercoiled plectoneme-like structures that can transition throughout the TAD but that cannot pass through boundaries, e.g., because boundaries are physically tethered. Simulations show that such structures can lead to TAD-like structures as detected by Hi-C [79, 80]. However, whether such structures are present in mammalian genomes at the level of hundreds of kb is unknown. Alternative possibilities do not directly involve boundaries themselves and include roles of long-range interactions within TADs in stabilizing these domains, but any model must include mechanisms by which such long-range interactions display directionality so that interactions across TAD boundaries are disfavored [81]. Whatever the model, boundaries are likely to be key factors that determine TADs. Indeed, deletion of a boundary leads to increased interactions between adjacent TADs [68].

Recently, several high-resolution chromatin interaction analyses have revealed additional domains and structures embedded within TADs [74, 76, 82]. Such “sub-TAD” structures are cell type-specific and may well represent another nested type of domain, but it seems more likely that structural differentiation within TADs is directly related to specific looping interactions between resident functional elements.

#### Chromatin looping in the interphase nucleus

Long-range gene regulation can involve direct physical interactions between promoters and distal regulatory elements [30, 83, 84]. Large, multi-Mb, chromatin loops have been detected by microscopy. For instance, in flies looping between two heterochromatic domains located on chromosome 2, chromatin loops have been directly visualized by FISH [85]. In addition, classical nuclear extraction methods to identify scaffold- and matrix-attached regions suggest that chromosomes form series of loops with their bases attached to nuclear structures (e.g., [38, 77, 78]). Such experiments have led to the general notion that chromatin loops are abundant and can be dependent on transcriptional activity of loci. Chromatin looping has also been seen by electron microscopy of DNA-protein complexes. For instance, looping was observed between specific loci, mediated by their bound proteins [86]. However, direct visualization of looping interactions between a specific enhancer and its target gene(s) has remained difficult due to the fact that enhancers and their target promoters tend to be located relatively close to each other in the genome (separated by at most several hundred kb and rarely more than 1 Mb). The resolution of light microscopy and the size of the probes that is required have made detection of loops at that length scale difficult.

The application of 3C-based assays has facilitated the detection of looping interactions at higher resolution (kb) and genome wide. Initially, looping interactions were identified and studied in loci of interest such as the alpha- and beta-globin loci [26, 87–89]. Increasingly, more comprehensive chromatin interaction analyses (3C-seq, 4C, 5C, T2C, Capture-C, and Hi-C) are being used to map looping interactions throughout the genome [49, 50, 73, 90–94]. From these studies, several general principles are emerging. First, looping interactions are very common and occur most frequently among and between active gene promoters, active enhancers, and sites bound by CTCF (e.g., [27, 49, 72, 93, 95]). Second, many of these looping interactions are directly implicated in gene activation. Promoters tend to interact with several (~2 to 4) distal regulatory elements [27, 95]. Third, most of these interactions occur over 10 to 200 kb, and only very rarely over longer genomic distances. It is noteworthy that this loop size is in accordance with previous estimates of interphase loop size using entirely independent methods (e.g., [96]). Combined with the observation that TADs are functional domains for enhancer action, it has been proposed that looping interactions between genes and distal regulatory elements occur mainly within TADs (e.g., [6, 70, 71, 97]). Consistently, >70% of looping interactions detected by Sanyal et al. are between sites located within the same TADs [27]. Shen et al. also reported a significant enrichment of promoter-enhancer pairs, predicted based on correlated activity across cell types, within TADs [98].

When interpreting looping interactions detected by 3C-based methods one needs to be aware of the fact that such associations may not be direct locus-locus interactions [7]. Significant proximity can also be obtained by association of pairs of loci to a common nuclear component such as the nuclear envelope, transcription factory, etc. [99, 100].

It is important to point out that specific long-range interactions can also occur between TADs and even between chromosomes. Such longer-range interactions were originally discovered by imaging approaches, e.g., between blocks of active domains [101] or heterochromatic regions [85]. These interactions, as detected by 3C-based methods, tend to be of low frequency especially for interchromosomal interactions, reflecting the fact that these interactions occur in only a small subset of cells (discussed in [6]). Although these interactions may impact gene expression in the few cells in which they occur [102], roles for such interactions may be more related to nuclear organization in general than to gene regulation (see below, and see [6] for a detailed discussion of intra- and inter-TAD interactions and their role in gene regulation).

### Differences in interphase chromosome folding between cell types

Many of the DNA elements involved in the spatial organization of chromatin display cell type-specific activity, whereas others are more general: enhancers are particularly cell type-specific and promoters are bound by polymerase more generally [23], while many of the CTCF sites are rather stably bound across cell types [103]. Looping interactions between promoters and enhancers are, therefore, also very different between different cell types, reflecting state-dependent gene expression patterns [27, 30].

A- and B-compartments are also cell type-specific [50]. This is again a reflection of the fact that different cells express different sets of genes and thus have different regions of their genomes in active and open conformations. This is also manifested in the fact that association of some loci with the nuclear lamina can be cell type-specific and related to the expression and/or chromatin status of the locus. For instance, during cell differentiation, loci move away from the lamina as they become active (e.g., [104]); a classic example is the activation of the IgH locus [105]. In hematopoietic progenitors, the locus is in a closed state, transcriptionally silent, and associated with the nuclear envelope. Upon differentiation of the cells into pro-B cells, the locus becomes activated, changes chromatin status, and is found in the nuclear interior.

TADs are distinct because it is the only organizational feature that displays low variability between cell types. It has previously been proposed that this feature of TADs makes them structural building blocks and places them in a central position in the hierarchy of the 3D genome [6, 70]. Their internal organization is cell type-specific and related to looping interactions that drive gene regulation. TADs themselves assemble into higher order structures, such as A- and B-compartments that are composed of different groups of TADs in different cell types depending on the transcriptional and chromatin status of the TADs [50, 106].

### Changes in chromosome folding during the cell cycle

Chromosomes change their appearance dramatically during the cell cycle (Figure 1). During prophase chromosomes become increasingly condensed and individualized, suggesting that many organizational aspects of the interphase chromosome conformation are lost. During metaphase chromosomes form linearly organized structures and transcription mostly ceases. DNA staining reveals that mitotic chromosomes display a largely cell type-invariant banding pattern [107]. Importantly, immunofluorescence studies show that metaphase chromosomes remain composed of a series of domains that differ in chromatin status [108]. For instance, large domains enriched in histone modifications associated with active chromatin alternate with domains displaying features of inactive chromatin. It is tempting to propose that such domains correspond to loci located in A- and B-compartments in interphase. It is important to note that the banding pattern of metaphase chromosomes is largely invariant and possibly directly related to differences in base composition along the chromosome, whereas A- and B-compartments and domains of histone modifications along chromosomes in interphase have a cell type-specific component.

Recently, 5C, Hi-C, and synchronous cell systems were employed to study chromosome conformation throughout the cell cycle [109]. These studies clearly confirm that many structural features described above for non-synchronous cells are specific to interphase, and in particular G1. In highly pure G1 cultures one can detect very prominent A- and B-compartments and TADs. This study did not analyze chromatin looping between promoters and enhancers, but it is reasonable to assume these interactions occur in G1, when the genome is actively being transcribed.

Interestingly, in mitotic cells both A- and B-com-partments and TADs are undetectable. Given that mitotic chromosomes are mostly transcriptionally silent and that many (but not all) transcription factors are dissociated from chromatin [32, 33, 35], it is likely that promoter-enhancer looping interactions are also absent. Higher resolution studies are required to address the fate of such looping interactions during mitosis in more detail.

The fact that A- and B-compartments are absent in mitosis may not be too surprising because chromosomes form linear sausage-like structures that do not accommodate the long-range homotypic intra- and interchromosomal associations that define compartments in interphase. In the context of the critical structural role that has been ascribed to TADs [6, 70], it is intriguing that these structures are also not maintained in mitosis. Thus, whereas TADs are rather stable across cell types, they are not stable during the cell cycle.

### Metaphase chromosome organization: a universal structure is formed

The organization of mitotic chromosomes has intrigued many biologists over the decades. Microscopic studies, as well as biophysical analyses with purified mitotic chromosomes, has led to various models for their internal organization [17, 18, 110–115]. These different models have been extensively discussed in several excellent reviews (e.g., [17, 18]). These models fall in two broad categories: one class of models proposes hierarchical folding of the chromatin fiber into increasingly higher order structures [17]. For instance, a 10 nm chromatin fiber can fold into a 30 nm fiber, then into a 100 nm fiber, and so on. Other hierarchical models can include hierarchical looping or a series of rosettes of rosettes. In the second class of models, mitotic chromosomes are composed of a series of loops that are attached to a central chromosome axis. Some models contain features of both. For instance, models proposed by Belmont et al. contain an axial core of the chromosome to which an irregularly packed fiber of various thicknesses is attracted [115]. Other models propose mitotic chromosomes fold as more disorganized networks, without hierarchical coiling and without a rigid proteinaceous axis [116]. Over the years, observations supporting one or more of these different classes of models have been made. Below, some of these proposed structures are outlined, followed by a presentation of how chromosome conformation capture experiments have contributed to testing, partially unifying and further refining these models.

#### Helical and hierarchical models

Initial microscopic observations indicated that mitotic chromosomes are composed of thick rods made up of tightly but irregularly folded fibers that can subsequently coil to form thicker condensed chromatids [117]. Since then, several authors have proposed a variety of models in which mitotic chromosomes are organized as folded fibers with different levels of coiling. For instance, Bak and Crick performed electron microscopy on purified and partially unfolded chromosomes and proposed that they fold as a hierarchy of helices to form a supersolenoid structure [110]. In further support of hierarchical coiling, fibers of varying thickness can be detected in fixed preparations (e.g., [113]). Combined with observations of non-reproducible radial positioning of loci inside mitotic chromosomes [118], this has led to strong support of several hierarchical features of mitotic chromosomes that are not always explained by classic loop-axis models (below). Extensive work by the Belmont laboratory also showed that mitotic chromosomes are folded as an irregularly condensed fiber with varying thicknesses, but those data also indicate that a strictly ordered hierarchy of increasing levels of coiling is too simplistic [113]. More recently Belmont et al. proposed a hierarchical folding – axial glue model, where the chromatin fibers folds hierarchically, but without strict order, into higher order fibers. An axial glue, consisting at least of condensin, then organizes a longitudinal chromosomal core of cross-linked chromatin [115].

#### Loops-axis models

Electron microscopy and immunofluorescence experiments have indicated the presence of a central axis that runs along the center of mitotic chromosomes. This axis is composed at least of topoisomerase II and condensin [114, 119, 120]. Swelling of mitotic chromosomes by removal of histones revealed chromatin loops that emanate from a dense axial structure [111]. Careful analysis of these loops allowed measurement of their length, which was estimated to range from 30 to 90 kb, with an average of around 80 kb [111, 121]. Further support for an axial structure to which DNA loops are attached comes from nuclease treatment of purified mitotic chromosomes followed by electron microscopy. After this treatment, scaffolds with the size and shape of the mitotic chromosome core were observed [121]. These observations led to the radial loop model where the mitotic chromosome is formed by a linear axis to which chromatin loops of ~80 kb are radially attached. This loop-axis structure can then coil to further shorten the chromosome, e.g., as observed in Boy de la Tour et al. [122].

One prediction of this class of models is that there may be specific sequences, spaced throughout the genome, that act as scaffold attachment regions (SARs). Indeed, AT-rich sequences have been identified by their association with scaffold preparations [123–126]. Consistent with this model, mitotic chromosomes can display AT-rich sequences lined up as a queue along the core of the mitotic chromosome [127].

#### Other observations and models

As already described above, several models contain features of both hierarchical folding, the presence of chromosome axes or cores, and chromatin loops. A quite different type of model was proposed by Poirier and Marko [116]. These authors performed biophysical measurements of chromosome elasticity and the effects of DNA digestion. These important studies led the authors to propose that no mechanically continuous proteinaceous axis is present, and that mitotic chromosomes are composed of a DNA meshwork stabilized with regular cross-links. They estimated these cross-linked to occur every 10 to 20 kb.

#### Chromosome conformation capture analysis of mitotic chromosomes

5C and Hi-C data of mitotic chromosomes allowed a re-assessment of some of these different models [109]. The 5C and Hi-C data of mitotic chromosomes revealed several striking features. First, as outlined above, any locus-specific features, very prominent in interphase, are absent in mitosis [109]. Second, Hi-C analyses of mitotic chromosomes from three different cell types did not reveal any cell type-specific features. Third, the relationship between interaction frequency and genomic distance between loci decays very slowly up to 10 Mb, but then drops precipitously. This contrasts with the G1 pattern that displays several regimes that are probably related to the hierarchy of compartments described above [6, 50, 109, 128]. Thus, in mitosis, a different and locus-independent conformation is formed.

The 5C and Hi-C data do not readily indicate the 3D arrangement of mitotic chromosomes, as it is currently not known how to directly infer the ensemble of 3D conformations that is consistent with chromosome-wide interaction maps. However, polymer simulations can be applied to test whether specific models for mitotic chromosomes are consistent with observed data. Thus, polymer ensembles folded according to the features of each class of previously proposed models can be generated by simulation and then be used to determine which would produce interaction frequency patterns along the chromosome that are most consistent with experimentally observed chromosome conformation capture data. To do this, simulated ensembles of conformations were generated and then tested by simulating the Hi-C procedure to determine whether they would reproduce the two main features of the observed data: a locus-independent homogenous interaction map and the mitosis-specific decay of interaction probability vs. genomic distance. None of the regularly ordered hierarchical models reproduced the shallow decay of interaction probability for loci separated up to 10 Mb. Instead, in such models, interaction probability decayed very fast, presumably because loci do not readily mix with loci located farther away and that will be located in higher order levels of the hierarchical structure.

Meshwork models, as proposed by Poirier and Marko [116], and Nishino et al. [129], were not explicitly tested. However, simulation of random non-consecutive loops, which may resemble a disordered meshwork, did not yield models that accurately reproduced chromosome conformation capture data. However, more explicit simulations are required to explore the presence of a disordered meshwork of cross-linked chromatin.

Interestingly, formation of arrays of consecutive chromatin loops produced predicted chromatin interaction data that are consistent with experiment, supporting the presence of chromatin loops inside mitotic chromosomes [109]. From a series of simulations, the following conclusions were made. i) Chromosomes form arrays of loops; these loops must be consecutive, and models built as non-consecutive loops do not produce interaction data consistent with experimentally observed data. ii) Chromatin loop size is not fixed, but ranges from 80 to 150 kb (assuming a 10 or 30 nm fiber). iii) Loops are stochastically positioned, i.e., the sequences at the bases of the chromatin loops vary between cells in the population. It is important to note that this does not mean that loop positioning is completely sequence-independent: it is possible that a certain type of common sequence element is located at bases of loops, but that each cell picks a different subset of these sequences. iv) Models with and without a centrally positioned axis produced data that closely fit the experimental data. v) Cells must not only have mechanisms to generate these loop arrays, they must also have mechanisms to shorten these arrays to produce the typically short mitotic chromosomes. Such shortening can be observed cytologically during and after pro-metaphase. vi) Analyses of three different cell types showed that the way mitotic chromosomes are folded is invariant: no cell type-specific interactions are present, at least at the resolution of the current study (40 to 100 kb). Combined, these analyses indicate that mitotic chromosomes are folded as linearly compressed stochastically positioned consecutive loop arrays (Figure 1).

Satisfyingly, this model of a compressed stochastic loop array unifies many experimental observations that have been collected over the years. The model supports many aspects of the classic loop-axis/radial loop models pioneered by Laemmli et al.: the presence of an array of loops of around 80 kb as had been observed by electron microscopy, e.g., [111, 121]. The model derived from these chromosome conformation capture data and simulations displays variability at many levels. For instance, since loop positioning and size are variable, the model predicts highly variable radial localization of loci. This has indeed been observed [118], but had been interpreted in terms of hierarchical models. As stated above, it is important to point out that random loop positioning does not rule out that specific sequence elements are preferably found at the loop bases (e.g., SARs, as described by Laemmli et al. [125]): a different subset of such sequences could be found at loop bases in different cells in the population. Further, the chromatin loops in these simulations are highly disordered and irregularly condensed to fit inside the volume of a mitotic chromosome. This may be consistent with observations that chromatin fibers of different diameters can be seen in mitotic preparation [113, 130]. Similarly, in the most compact state, individual fibers themselves may become impossible to trace and a melt of nucleosomes is formed, consistent with tomography experiments [129]. Also, no regular or rigid axial structure was required to reproduce the chromosome conformation capture data by simulation: simulating a more variable path of the loop array also reproduced the observed chromatin interaction data. This is in agreement with a more diffuse chromosomal core observed by Belmont et al. [115] and the lack of a robust DNA-independent mechanically continuous proteinaceous axis as shown by Piorier and Marko [116].

The compressed stochastic loop array model is based on a series of chromatin loops, as in the loop-axis models described above. Yet, the model also reproduces some findings that had been interpreted in terms of more hierarchical models. For instance, FISH experiments suggested that mitotic chromosomes form large 250 nm gyres [115]. Perhaps somewhat unexpectedly, it was shown that such observations are also consistent with the compressed stochastic loop array model [109]. The high level of variability that is present in the stochastic loop array model can lead to chromosomes that have an irregular packing, again as observed by Belmont et al. [115].

Many details of mitotic chromosome folding are still lacking. Higher resolution 5C/Hi-C and imaging experiments will provide more insights into these iconic structures, and may lead to new ideas for how they are formed.

### Cell type-invariant mitotic chromosome folding: implications for epigenetic inheritance

Mitotic chromosomes are thought to retain an epigenetic memory of which genes and regulatory elements are active or inactive in the corresponding cell type [31, 35]. Such memory must occur while many chromatin-associated factors, including RNA polymerase II and many transcription factors, dissociate from chromosomes and, concomitantly, chromosomes loose their cell type-specific long-range interactions and 3D folding. Clearly, 3D structures, e.g., cell type-specific chromatin looping interactions and chromosomal compartments are not themselves epigenetic features that are inherited. Yet, in the subsequent G1 stage, a cell type-specific chromosome and nuclear organization readily reforms. As outlined above, TADs are mostly tissue-invariant, and therefore it is possible that their re-establishment after each cell division is part of a canonical pathway of chromosome re-folding in early G1 shared by all cell types. However, the internal folding of TADs, and the assembly of TADs into higher order A/B-compartments, is cell type-specific. Here, it is proposed that local epigenetic memory, or bookmarking, of TAD boundaries and of the locations of previously active genes and regulatory elements suffice for rebuilding the global 3D genome.

### Epigenetic inheritance of locally encoded instructions for 3D genome folding

It is believed that active promoters and regulatory elements somehow become bookmarked in mitosis, although it is not known in detail how this is implemented. Possibly, several key chromatin components remain associated with (a subset) of elements (e.g., [33, 34]). Alternatively, no binding is required, but relevant DNA elements simply have to remain accessible so that factors can rebind in the next G1 [32]. Where and how is cell type-specific information related to chromosome folding stored? Could this occur through a similar process as cells use to remember which genes need to be active? Could this even involve the same DNA elements? If so, what does that mean for the process by which the 3D genome forms? Here, it is argued that cell type-specific instructions for 3D folding, e.g., enhancer-promoter pairing, compartments, etc., are encoded in local properties of chromatin and that no specific memory for higher order 3D folding is required. In this model, outlined below, most aspects of genome folding in interphase cells are driven by self-assembly guided by these local instructions. This will lead to reproducible local 3D interactions within TADs, but also to increasingly stochastic higher order assemblies, as observed (see [6] for further discussion of stochasticity in genome folding). It is noted that self-assembly is not a new idea and self-assembly models for nuclear and chromosome organization (e.g., [4, 131–134]), and bookmarking of individual elements have been described before (e.g., [31, 32, 34]).

### Re-formation of cell type-specific chromosome organization in early G1 by local action and self-assembly

In interphase A/B-compartments, TADs, and chromatin loops between genes and regulatory elements define the 3D genome. During prophase many factors dissociate from chromatin and perhaps as a result of this many features of the 3D genome, including compartments and TADs, are lost (Figure 1). The cell then re-folds the genome into the mitotic stochastic loop arrays. Inside this structure, TAD boundaries as well as cell type-specific DNA elements, such as enhancers, remain marked either by specific proteins [33, 135] or simply by remaining in a nucleosome-free state. Thus, these elements remain marked, but do not affect the 3D folding of the chromosome. For instance, in this model, TAD boundaries remain marked, but have lost their ability to prevent mixing of adjacent chromatin domains. After anaphase, the mitotic chromosome folding machinery dissociates and proteins and complexes such as transcription factors and RNA polymerase re-associate to chromatin at the marked DNA elements. Loading of these complexes is then sufficient to drive cell type-specific chromosome folding through local action and stochastic self-assembly. The observation that promoter-enhancer looping occurs mostly within TADs and that groups of TADs assemble into A- and B-compartments directly implies that the process of 3D genome assembly occurs in a temporally controlled fashion (Figure 1). Specifically, given the central role of TADs in this hierarchy, their formation must occur first. Thus, re-loading of complexes at TAD boundaries and the imposition of TAD insulation must be a rapid and early event in G1. Perhaps this rapid re-loading is the result of mitotic bookmarks at TAD boundaries. Once TAD boundaries are established, promoter-enhancer complexes located within TADs can engage in looping interactions. As discussed before, these interactions are transient and dynamic and will occur throughout G1 to accommodate intra-TAD promoter-enhancer pairing in all cells in the populations [6, 30, 70, 71, 81, 97]. After TAD definition, groups of TADs self-organize into higher order assemblies (A/B-compartments). Self-assembly generally results in stochastic structures. Indeed, A/B-compartmentalization, as well as other higher-order features, such as chromosome territory positioning, association of domains with the nuclear lamina, etc., are known to be variable between otherwise identical cells and are not inherited through mitosis [136, 137]. Assembly of these higher order structures could be mediated by transcription factor complexes bound to chromatin, or simply by preferential clustering of chromatin domains that are similar in histone modifications. Patterns of several histone modifications are cell type-specific and are stable in mitotic chromosomes [108].

## Conclusions

This article reviewed the many studies performed over the years focusing specifically on the contributions of chromosome conformation capture that have led to important insights into the two ways cells fold their genome during the cell cycle. A model for mitotic transmission of folding instructions was then presented. The model implies that looping interactions between promoters and enhancers only require locally bound complexes, and that TADs are important for limiting which promoter-enhancer pairings occur. Finally, the self-assembly model for nuclear organization and the resultant high cell-to-cell variability at the scale of compartments suggest that these higher order structures are not involved in determining robust cell type-specific gene expression in all cells in the population. This proposal makes clear predictions related to the order of events in early G1 and the roles of specific DNA elements and protein machineries that can now be tested by using synchronous cell cultures, chromatin interaction, and imaging methods, as well as more recently developed genome engineering approaches.

## Abbreviations

3D: Three-dimensional
3C: Chromosome conformation capture
4C: Circularized chromosome conformation capture or Chromosome conformation capture-on-Chip
5C: Chromosome conformation capture carbon copy
CD: Chromosomal domains
FISH: Fluorescence In Situ Hybridization
SAR: Scaffold attachment region
TAD: Topologically associating domain.
